# Online assessment in undergraduate medical education: Challenges and solutions from a LMIC university

**DOI:** 10.12669/pjms.37.4.3948

**Published:** 2021

**Authors:** Syeda Sadia Fatima, Romana Idrees, Kausar Jabeen, Saniya Sabzwari, Sadaf Khan

**Affiliations:** 1 Syeda Sadia Fatima, PhD. Department of Biological and Biomedical Sciences, Aga Khan University, Karachi, Pakistan; 2 Romana Idrees, FCPS. Department of Pathology and Laboratory Medicine, Aga Khan University, Karachi, Pakistan; 3 Kausar Jabeen, FCPS. Department of Pathology and Laboratory Medicine, Aga Khan University, Karachi, Pakistan; 4 Saniya Sabzwari, MD. Department of Family Medicine, Aga Khan University, Karachi, Pakistan; 5 Sadaf Khan, MD. Department of Surgery, Aga Khan University, Karachi, Pakistan

**Keywords:** E-assessments, Online teaching and learning, assessments, Medical education, Virtual learning environment

## Abstract

**Background and Objectives:**

The Covid-19 pandemic has caused large-scale disruption in almost all educational programs across the world. Planning and rapid implementation of assessment through an online format presents the next set of novel challenges that must be addressed by academic administrations across the globe.

**Methods:**

This cross-sectional study was conducted between March to August 2020 at the Aga Khan University Medical College. Two hundred medical students of year 1 and 2 participated in the study. We describe the planning, processes, and outcomes of online assessments using video communication platforms conducted at a private university in Pakistan. Standardized protocols were written and piloted, extensive training of student, proctors and staff for preparation and conduct of online assessments were developed. Feedback was recorded after each session and suggestions were incorporated in subsequent high-stakes assessments.

**Results:**

A total of three pilot assessments were conducted to identify issues and process refinement. Commercially available lockdown browser and ZOOM were used in the first pilot; 80% of the class was unable to launch lockdown browser and laptops required repeated reload/reboot. For the second pilot assessment, University’s VLE page & MS Teams was trailed. Issues with internet connectivity, VLE page slowdown, and suboptimal recording feature in MS Teams were identified. For the final pilot assessment, phased launching of VLE page with single test item per page was implemented with success. The students reported that attempting the online exam on VLE with ZOOM support was user friendly. Ninety percent of the class was supportive of the continuing with the online assessments.

**Conclusion:**

In order to device an effective protocol for e-assessments conducting multiple trial runs, and incorporating feedback from all stakeholders is a necessity.

## INTRODUCTION

The Covid-19 pandemic has had a major influence on almost all aspects of life across the globe.^1^,^2^ Not far behind was the impact on educational systems across the world.^3^ These systems have traditionally been based on face-to-face interaction and the pandemic situation has demanded rapid adaptation and improvisation on the part of governing bodies, educational institutions, teachers, students, and parents.^4^-^6^ Failure to modify established methods of teaching and assessment would result in significant long-term impact on the educational trajectory and/or potential career progression for youth across the world.^7^

The millennial students expect their online teaching and learning experience to be intellectually stimulating, allowing for meaningful interactions, and proximate feedback.^8^,^9^ At the Aga Khan University Medical College, Pakistan, we initiated online teaching for the pre-clinical students shortly after imposition of a country-wide lockdown. As the COVID situation escalated across the country, it became imperative for us to plan the next steps for student progression through the curriculum. In addition, the higher education commission (HEC) also gave directives and issued some basic policy guidelines to conduct assessments. Therefore, we sought to develop and compare processes and outcomes of online assessments that could help all educationists to plan and implement e-assessments in their settings.

## METHODS

This cross-sectional study was conducted between March to August 2020 at the Aga Khan University Medical College. Two hundred medical students of year 1 and 2 participated in the study. The Institutional Ethical review Committee gave approval for the study (Approval No: 2020-4780-11438, Dated: July 15, 2020).

### Protocol development and Pilot Testing

University’s Virtual Learning Environment (VLE) (https://vle.aku.edu/)^10^ was used for conducting these assessments [3 pilot non graded and 2 summative]. Exam questions were retrieved from the University’s Question Bank. Items were selected based on the learning objectives covered during the module and item performance metrics i.e. difficulty level and discrimination index. The examination was constructed with a C2 (Objectives of Interpretation) /C3 (Objectives of problem solving)/ C1 (Objectives of recognition & recall C1) ratio of 70:20:10.^11^ Test items included MCQ, EMQ, Drag and Drop, and Short answer questions based on the modules being assessed namely Endocrine Reproduction, Gastrointestinal and Nutrition, Infection and Immunity, Respiration and Circulation. To deter cheating, two sub-types of randomization were utilized while formatting the assessment: 1) all students completed the same assessment, with multiple item order and answer option randomizations, and 2) the items comprising the assessment were randomly divided into two parts. The order of the two parts was random for each student. Students were able to navigate between items assigned to one block, but could not navigate between blocks. Students were required to score a grade of 55% to successfully clear the exam. Respondus (http://www.respondus.com)^12^, was used for the first pilot which functioned well on campus supported by proctoring via ZOOM.

For the second pilot assessment The University’s VLE page supported by proctoring via MS Teams. The third and final pilot examination, Zoom was used for the proctoring video call and recording and VLE was used as exam portal. The exam was set up so that each page displayed a single test item. If there was an issue with connectivity, the student would know as soon as he/she attempted to progress to the next page, thus reducing the risk of ‘losing’ their responses. We opted for phased launching of the VLE assessment page in order to avoid heavy traffic and browser failure. Irrespective of the induction time, each student received the same amount of time to attempt the quiz.

To ensure that students reflected on the implications of dishonesty, a short integrity statement was composed in consultation with the Examination and Promotion committee, and had to be ‘agreed’ to by the student before the assessment was launched. The time for the e- assessment was set for a shorter duration as compared to the conventional face to face paper, based on available literature.^13^,^14^ Specific standardized protocols for the preparation and conduct of the pilot formative assessments were developed for students, proctors, and administrative support. These along with the e-assessment page link, enrollment key, and video streaming links were shared one week prior to the exam via emails and VLE notification. The instructions were comprehensive and included details related to bandwidth requirement, camera placement, joining time, exam time and duration, contingency planning in terms of power outage or internet connectivity issues, contact persons’ phone numbers, proximity check procedure etc. Special adaptations for individuals needing extra time, including dyslexic students or those facing power outage/internet connectivity issues were also planned. Training sessions with students, proctors and administrative staff were conducted before the first pilot. These sessions served as a dry run to identify issues that may not have been anticipated. In addition, a tutorial video, detailed instructions, and a script for online invigilation was developed for the proctors. A brief communication was sent to the parent body to update them about the process of e-assessments.

The exam was delivered online to the relevant cohort of students at a prescribed time. Eleven and twelve teams were created respectively for year one and two students. Each team had nine students under supervision of one proctor. One administrative assistant monitored two virtual rooms and on-site IT support was available for the entire duration of the exam. A pictographic list of students assigned to each group was provided to the proctors. A WhatsApp group was created with the proctors, faculty leads, IT support, and admin support to facilitate communication and rapid response to SOS calls during the exam. Proctors performed a detailed proximity check for each student (where the student would show his/her room, desk, laptop and any paper or material on desk for approval) and reported activity completion on the WhatsApp group. Once all stations reported activity completion, the password to launch the assessment was shared with proctors, who relayed it to the students. A post hoc analysis was conducted after the second and third pilot exam to assess its reliability and validity compared to the face to face exams.

### Data Collection

Data collection was conducted in real time during each pilot run where a checklist was provided to the invigilators to record any issues and challenges faced for that particular pilot. Student feedback was obtained through Microsoft Forms on a likert scale (where items were rated as 0-5 Strongly Disagree; Disagree; Neutral; Agree; Strongly Agree) after the online exam. The items focused on the accessibility of VLE, ZOOM, internet issues, and support etc. An option to add open ended comments was also provided. The form was pre tested on 20 year 3 students, who were not participating in the study (Cronbach’s alpha 0.86).

The data was presented by calculating an average score with standard deviation for each item. The response rate was 100% for this activity. Further, after each pilot an in depth discussion was conducted with all stake holders by an independent researcher who was not involved with the teaching learning and assessment. These sessions were recorded and transcribed later by the researchers. The responses were collated at the end and were grouped in themes of Pros and Cons for the online assessment.

## RESULTS

Two hundred students (Male 106: Female 94) from Year 1 and 2 MBBS, age 20 ± 1.85 year studying at Aga Khan University Medical College participated in the study.For the first pilot; lockdown browser failed to launch for 80% of the class. There were additional issues including laptops slowing down, loss of connectivity, and the need to reload/reboot repeatedly. It was identified during the feedback cycle that once the lockdown browser was launched, students could not access the home button to exit the program unless they submitted that examination attempt. This was complicated by laptops ‘freezing’. These issues were related mostly to the variability of bandwidth and connectivity across the different geographic locations such as rural versus urban areas that our students were based in. All these issues lead to cancellation of this pilot assessment.

A different set of issues were identified for the second pilot. Even though the MS Teams call screen displayed nine students, it was only able to record four participants at a given time. The recording screen kept switching between participants, probably triggered by movement or sound. This was a considerable impediment to proctoring the exam as endorsed by the facilitators during the feedback. Another issue identified was the delayed loading of the VLE page when all 100 students signed on at the same time. Despite these issues, students were able to complete the exam.

**Fig.1:**
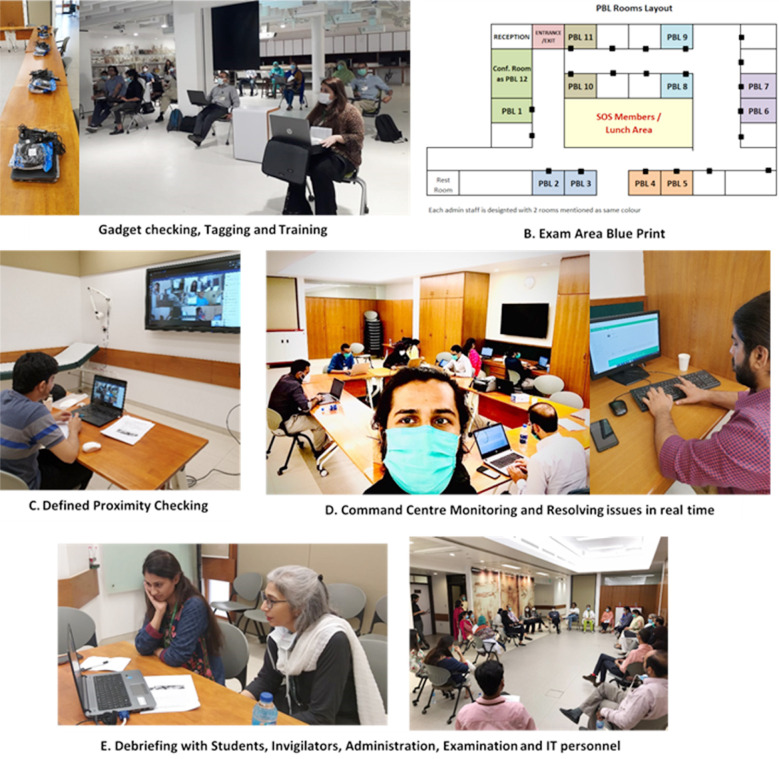
Proximity checks conducted on ZOOM and exam delivered using VLE/MOODLE (password protected quiz). Only students who cleared the proximity checks were given the password to attempt quiz. Image shows exam process and setup in a dedicated room. All proceedings were recorded.

No major issues were observed in the third pilot. All students were able to attempt the questions easily through VLE, proctors were able to watch each student and record the full proceeding without any issue. All students in each virtual room were visible and monitored throughout the exam period. This protocol from the third pilot was then implemented for the summative examinations with great success Fig.1. The overall reliability and validity of this exam was comparable to the conventional exam result (reliability of 0.789 and validity of 0.890). Further, the average student scores and class average scores also remained within one standard deviation of their previous face to face scores.

The student responses is summarized in Table-I. The feedback was promising and they reported that attempting the online exam on VLE with ZOOM support was user friendly, as VLE was able to save the work in case anyone faced internet connectivity issues. Online proctoring was not intimidating and was helpful in terms of solving issues in real time. Ninety percent of the class was supportive of the continuing with the online assessments.

**Table-I:** Likert scale score for Student Feedback on Online assessment.

Feedback Questions	Score Out of 5
Attempting online assessments on Virtual learning environment (VLE) and ZOOM was easy	4.50 ± 0.52
Online assessment questions were linked with the learning outcomes taught during the module	4.61 ± 0.43
The performance in Online assessments guided me to improve my learning and or study habits for future	4.59 ± 0.35
I was able to reach out to faculty in case I faced any issues in real time	4.54 ± 0.44
The time allotted for the tests were sufficient	4.56 ± 0.52
I faced internet connectivity issues during the assessment	3.3 ± 0.12
VLE was able to save my work in case I faced any internet issues	4.16 ± 0.15
I attempted the quiz on my own without using teaching aids	4.32 ± 0.26
Visual proctoring was effective in making the exam reliable	90% Yes
	10% No

***Where:*** 0 Strongly Disagree, 1 Disagree, 2 Neutral, 3 Agree, 4 Strongly Agree.

The summary of pros and cons identified by the students, proctors and administration during the feedback cycles of pilot tests is shown in Table-II. Majority faculty supported the virtual examination environment. They found it easier to manage, observe students in depth, identify any irregularities and help students in case of any technical issues. Further, the commented that essay paper marking was much easier on the VLE page versus hand written papers, mostly due to the nature of responses being typed and not handwritten. They also praised the support from the administrative team command center during the exam duration, especially regarding making resources available and real-time support services using multiple channels of communication.

**Table-II:** Pros and Cons from the Online Assessment Pilots Runs.

Online Proctored Exam Summary		

Students	Faculty	Administrative Staff/IT
**PROS**		
Immediate Feedback	Results can be immediately reviewed by an exam board	Easy to scan a large class
Greater tracking and transparency	Reduce marking loads	Recording available for review later
e-formative: Access their individual scores and marks more rapidly and confidentially, and see their aggregated assessment performance over time to help them manage their own study and performance	Support a wider range of questions and interactions	Secured against cheating
	Proctored; under controlled environment	
**CONS**		
Equipment overheating, or charge required	Formatting limitations	Provide equipment, invigilation and assurance of candidate identity and security
Computers/laptops not compatible with software’s	Technical failure	Provide Training to both faculty and student
Connectivity	Bandwidth issues when using images and video	Create Teams and monitor
Electricity/power failure	Longer duration of exams and screen time	Create unique passwords for exams
Bandwidth issues when using images and video		Secure location for faculty to invigilate due to COVID and social distancing
Background noise if all microphones are kept unmute		Lockdown browser was incompatible with many systems

## DISCUSSION

In this COVID induced challenging environment, programs were forced to make quick changes towards delivering classes online and make decisions regarding high-stakes assessments online. In addition, student apprehensions about progression in the academic year and examinations during online education was also rising.^15^ In response, many medical schools adapted a heterogeneous approach either delaying the exams or using aegrotat scores from previous summative and formative exams in order for the students to progress or graduate. Whereas some have used open book assessments for both clinical and preclinical medical students.^16^,^17^ At Aga Khan University, we decided to modify examination to be completed under proctoring from home via an online system. One benefit of offering examinations online was the significant student engagement and improved attendance during the online teaching learning sessions as supported by other studies.^18^-^20^ Additionally, our results show that pilot testing was a good way to simulate the virtual environment of the examination. The prior experience of students and staff with the pilots played a large role in the ease with which the assessments were conducted. Students appreciated the quality of e-assessment, and the support and assistance provided to them during the entire process.

The feedback received from faculty and students after these exams was very promising. Some of the student’s responses were as follows “*Just wanted to thank you all for the effort to make sure that we have a smooth summative exam. Thank you for listening to our feedback and solving our problems. Online summative exams are better than the real ones!*” Another student reported that “*It was a strange feeling to give an end of module exam from the comfort of my home. Yet, the quality of questions and the overall exam setting was at par to the onsite setup. The exam tested my knowledge and closely simulated the reality of having given the exam in person*”.

Furthermore, the reliability and validity assays for these exams were also at par to any face to face examination conducted. This aspect is considered most important while assessing the success of any high-stake exam.^21^-^23^

### Limitations of the study

The study is limited in a way that even though we have a diverse set of students living in remote parts of the country; most are financially secure. Despite this limitation, our experience from an LMIC University, with a student body distributed over multiple rural and urban locations, and widely variable technologic capability may help other institutes in the same geographical area to adapt and implement in their setup in this crisis.

After the successful implementation of the third pilot exam, a summary of events was presented to the institutional Curriculum Committee and Examination and Promotion Committee for discussion and approval. The online assessment protocol was approved and online summative examinations at the University were conducted successfully as a routine.

## CONCLUSION

The protocol followed in this study gives a foundation for medical universities to set standard operating procedures for online assessments in medical education.

### Author`s contributions:

**SSF, RI, KJ, SZ, SK** conceived and designed the protocol, conducted all three pilots, gave intellectual inputs during the process and wrote the manuscript.

All authors participated in drafting the article or revising it critically for important intellectual content; and gave final approval of the version to be submitted and any revised version.
